# Case Report: Identification of a *de novo* Microdeletion 1q44 in a Patient With Seizures and Developmental Delay

**DOI:** 10.3389/fgene.2021.648351

**Published:** 2021-05-20

**Authors:** Yiehen Tung, Haiying Lu, Wenxin Lin, Tingting Huang, Samuel Kim, Guo Hu, Gang Zhang, Guo Zheng

**Affiliations:** ^1^Department of Neurology, Children's Hospital of Nanjing Medical University, Nanjing, China; ^2^Department of Anesthesiology, Emory University School of Medicine, Atlanta, GA, United States

**Keywords:** 1q44 microdeletion syndrome, copy number variation, whole exon sequencing, seizure, developmental delay

## Abstract

**Objective:** 1q44 microdeletion syndrome is difficult to diagnose due to the wide phenotypic spectrum and strong genetic heterogeneity. We explore the correlation between the chromosome microdeletions and phenotype in a child with 1q44 microdeletion syndrome, we collected the clinical features of the patient and combined them with adjacent copy number variation (CNV) regions previously reported.

**Methods:** We collected the full medical history of the patient and summarized her clinical symptoms. Whole-exome sequencing (WES) and CapCNV analysis were performed with DNA extracted from both the patient's and her parents' peripheral blood samples. Fluorescent quantitative PCR (q-PCR) was performed for the use of verification to the CNV regions.

**Results:** A 28.7 KB microdeletion was detected in the 1q44 region by whole-exome sequencing and low-depth whole-genome sequencing. The deleted region included the genes COX20 and HNRNPU. As verification, karyotype analysis showed no abnormality, and the results of qPCR were consistent with that of whole-exome sequencing and CapCNV analysis.

**Conclusion:** The patient was diagnosed with 1q44 microdeletion syndrome with clinical and genetic analysis. Analyzing both whole-exome sequencing and CapCNV analysis can not only improve the diagnostic rate of clinically suspected syndromes that present with intellectual disability (ID) and multiple malformations but also support further study of the correlation between CNVs and clinical phenotypes. This study lays the foundation for the further study of the pathogenesis of complex diseases.

## Introduction

Many disorders have been reported to be associated with copy number variation (CNV), especially intellectual disability (ID), developmental delay, epilepsy, and other neurodevelopmental diseases in recent years. We conducted molecular genetics detection on a patient who was suffering from refractory epilepsy and detected a 28.59 KB heterozygous deletion. The adjacent CNV regions (1q41q42,1q43) have been reported to be associated with epilepsy and intellectual disability (ID), while the 1q44 region has been less reported (Filges et al., 2010; Shimojima et al., 2012; Balak et al., 2018). The 1q44 region is located at the very end of the long arm of chromosome 1, so both interstitial and terminal deletions have been described (patients with terminal deletions seem to have a more severe volume loss in the brain as compared with patients who harbor interstitial deletions). The existing reports show that microdeletion of 1q44 leads to a phenotype that includes microcephaly, seizure, agenesis or hypogenesis of the corpus callosum, polydactyly, congenital heart defects, and severe developmental delay along with characteristic facial dysmorphic signs. Here, we describe a 1q44 microdeletion patient with a 28.59 KB deletion, which is the smallest missing fragment reported so far. The patient has seizures, facial dysmorphic signs, agenesis of the corpus callosum, and intellectual disability. Meanwhile, we reviewed and analyzed the clinical features and CNV regions of children with CNV deletion in region 1q44 to further clarify the correlation between the deletion region and phenotype.

## Material

The patient is a 5-year-old girl who presented with global developmental delay for 5 years and seizures for half a year. She is the only child of non-consanguineous parents and there are no similarly affected family members. The patient was an SGA (small for gestational age) newborn who was delivered at a gestation age of 41 weeks with a birth weight of 2.1 kg (<3rd on the Olsen growth chart). Our patient has suffered from global developmental delay since birth, she could not crawl until she was 1 and a half years old and finally achieved walking in the 2nd year of her life. At present, she can neither verbalize a complete sentence nor pronounce words clearly. Her PPVT (Peabody picture vocabulary test) reported the patient's intelligence quotient (IQ) score as 34, which indicated that she is intellectually disabled (ID).

The first convulsion occurred at 1 year and 9 months old. The initial event was triggered by fever. Numerous episodes of febrile seizure happened recurrently in the next 2 years. After a 1-year gap when she was seizure-free without any anti-epileptic drugs (AED), epileptic seizures occurred at 5 years and 5 months old. Anti-epileptic medication (topiramate) was given to the girl after her first episode of epileptic seizure which occurred 2 months ago. She was admitted to our neurologic department 2 months later as the medication did not improve her condition and even triggered an absence seizure.

**Figure 1:**
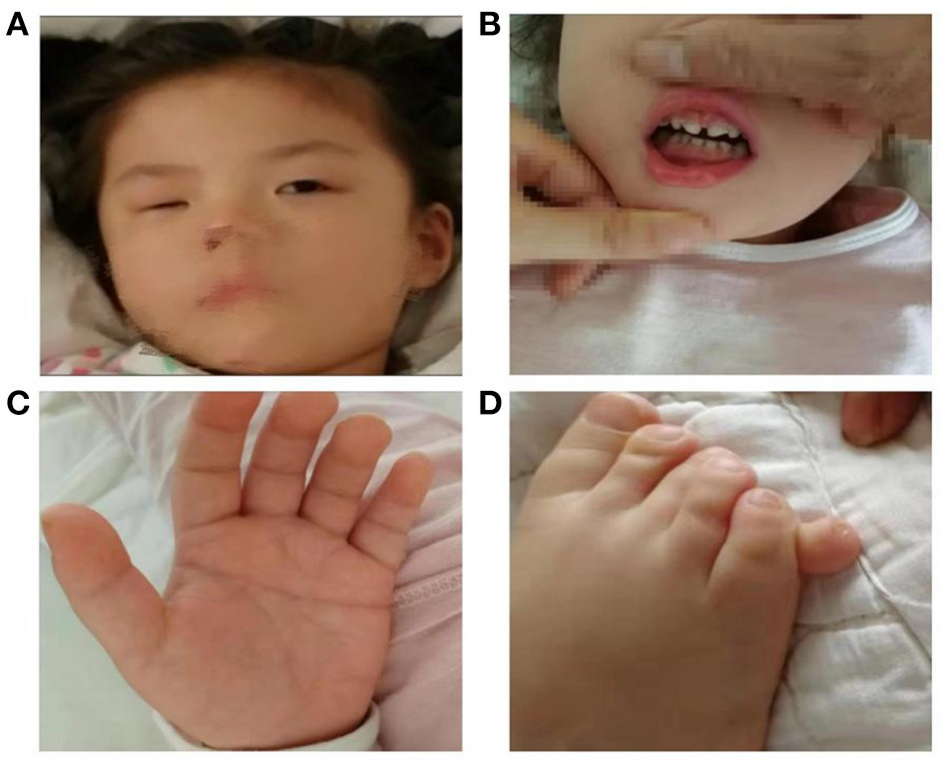
Female patient at the age of 5 years old presenting with facial dysmorphic signs including apparent asymmetry hypoplasia of eyes **(A)**, widely spaced front teeth **(B)**, Simian crease **(C)**, and polydactyly **(D)**.

On physical examination, facial dysmorphism was noticed including apparent asymmetry hypoplasia of the eyes (Figure 1A), left nystagmus, and widely spaced front teeth (Figure 1B). Her palmar crease was transverse on both hands (Figure 1C), and her right smallest toe was duplicated (Figure 1D). Her head circumference of 54.0 cm (>97th per China standard) and height of 107 cm (10th in the CDC growth charts) suggested short stature.

**Figure 2:**
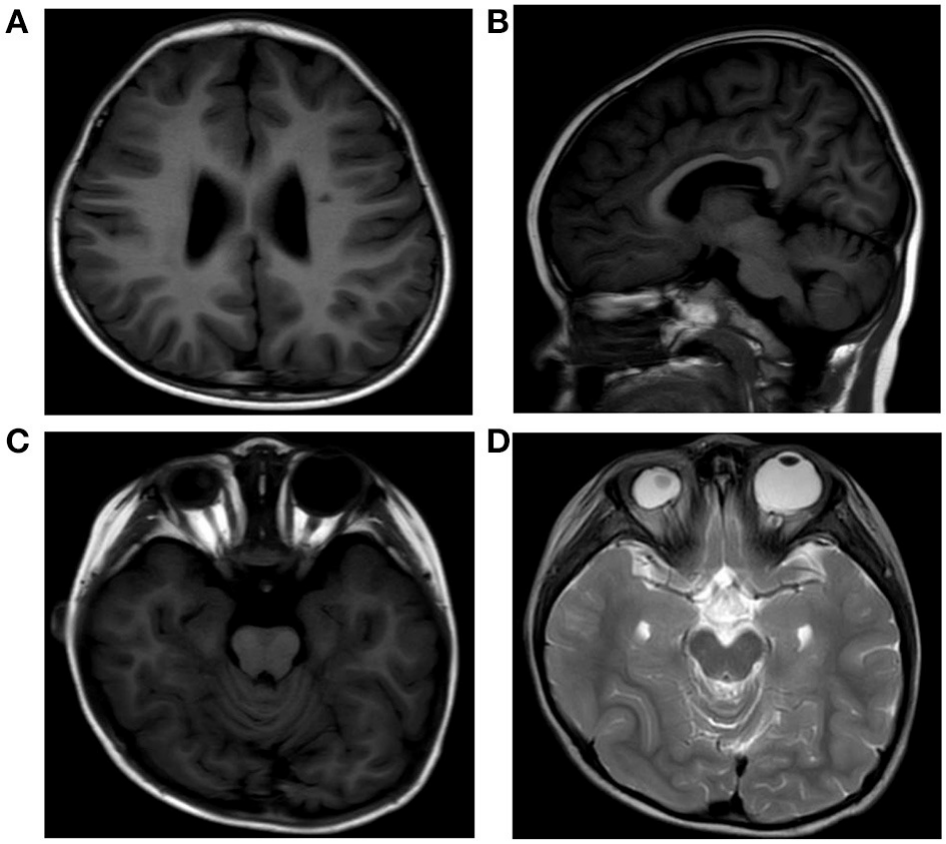
MRI of the patient's brain showing dilation of bilateral ventricles, a small patchy abnormal signal in paraventricular white matter **(A)**, hypogenesis of corpus callosum **(B)**, small right eye, and abnormal shape and signal of the lens **(C,D)**.

Investigations including conventional blood tests did not present any remarkable results. Analysis of organic acids in urine and acylcarnitine profile also revealed unremarkable results. Her brain magnetic resonance imaging (MRI) showed dilation of bilateral ventricles, a small patchy abnormal signal in paraventricular white matter (Figure 2A), hypogenesis of corpus callosum (HCC) (Figure 2B), small right eye, abnormal shape and signal of the lens, and slightly thinner optic nerve on the right side (Figures 2C,D). Electroencephalography (EEG) showed generalized a slow spike-and-wave complex, polyspikes, and slow activities, maximal in the frontal head regions.

## Method

### Genomic DNA Extraction

We sent the 2 ml of peripheral blood taken from the patient and her parents to Kaiumph Medical Laboratory. The genomic DNA was extracted using the blood genomic DNA extraction kit (QIAamp DNA Blood Mini Kit, Qiagen).

### Chromosome Microarray

DNA was extracted from peripheral blood. Then, the DNA samples were then processed by CMA using whole-genome bacterial artificial chromosome (BAC) microarrays, according to the protocol. The genomic coverage of this microarray platform is up to 1 Mb resolution. Data analysis was performed by algorithm fixed settings. The minimum number of microarray probes used for calling a copy number change was at least three contiguous BAC clones. Detected CNVs were compared to known CNVs in publicly available databases, and classified as clinically significant, likely benign, or as variants of uncertain clinical significance (VOUS).

### Whole-Exome Sequencing

Genomic DNA was extracted from the peripheral blood and quantified with Nanodrop 2000 (Thermal Fisher Scientific). The genomic DNA library was constructed by the NEXTflex Rapid DNA-Seq Kit (BIOO). Subsequently, the constructed libraries were captured by xGen Exome Research Panel v2 (IDT, Coralville, Iowa, USA) and sequenced for 10–12 GB by Novaseq 6000 (Illumina, San Diego, CA, USA). Alignment was performed by the Short Oligonucleotide Analysis Package (SOAP) aligner software. Snp_indel was called by GATK and annotated by ANNOVAR. Variants were filtered by minor allele frequency in normal population, variant function, and prediction conducted by SIFT, Polyphen2, and MutationTaster. The pathogenicity of variants was evaluated according to the American College of Medical Genetics and Genomics (ACMG) guidelines.

### Cap CNV Analysis

For the same batch of samples, bedtools-2.16.2 coverage was used to calculate the depth per exon, which was used as the minimum statistical unit. Due to the deviation of the actual amount of sequencing data among different samples, the exonic depth of each sample should be normalized by its sequencing quantity. In order to further correct the bias in the amount of sequencing data, the corrected depth was obtained by dividing by the ratio of the sample sequencing quantity and the batch average sequencing quantity. The depth ratio was obtained by the corrected sample depth over batch average depth, which was then used to judge whether there was a potential CNV for a single exon region. If the depth ratio <0.7, it was judged as a potential deletion, and if the depth ratio > 1.3, it was judged as a potential duplication. When the rate of abnormal depth ratio in this area in this batch exceeded 20%, it was judged as a low quality area and filtered. Screening criteria for candidate CNV regions: (1) for OMIM disease-related gene exonic regions, at least two consecutive exons with abnormal depth ratio are required; (2) for non-OMIM disease-related gene exonic regions, at least 10 consecutive exons with abnormal depth ratio are required. The candidate CNV regions were then annotated by DGV and Decipher.

### Sybergreen Fluorescent Quantitative PCR

Design primers for the COX20 and HNRNPU genes and housekeeping gene ABL1 contained in the microdeletion region were used as internal reference genes to design fluorescent quantitative PCR primers. The relative quantitative values (RQ values) of COX20 (forward, TGCCAAAGCACCCCAAAAT; reverse, CCAAAGCCAGCCACAACAGA) and HNRNPU (forward, CGAACGAATAAGGGATGAGTAAA; reverse, GAACAGAAAGGCGGAGATAAAA) genes were calculated with ABL1 (forward, CTAAAGGTGAAAGCTCCG; reverse, GACTGTTGACTGGCGTGAT) as the internal reference gene by ABI QuantStudio^TM^ 6 Flex fluorescence quantitative analyzer for statistical analysis. Fluorescent quantitative PCR to the expression of related genes in deleted regions on the genomic DNA taken from normal controls, the patient, and her parents was performed. The PCR reaction reagents were from Cowin Biosciences Company, and Shanghai Sangon Biotech Company synthesized primer sequences.

## Result

### Chromosome Karyotype Analysis and Chromosome Microarray Analysis

**Figure 3:**
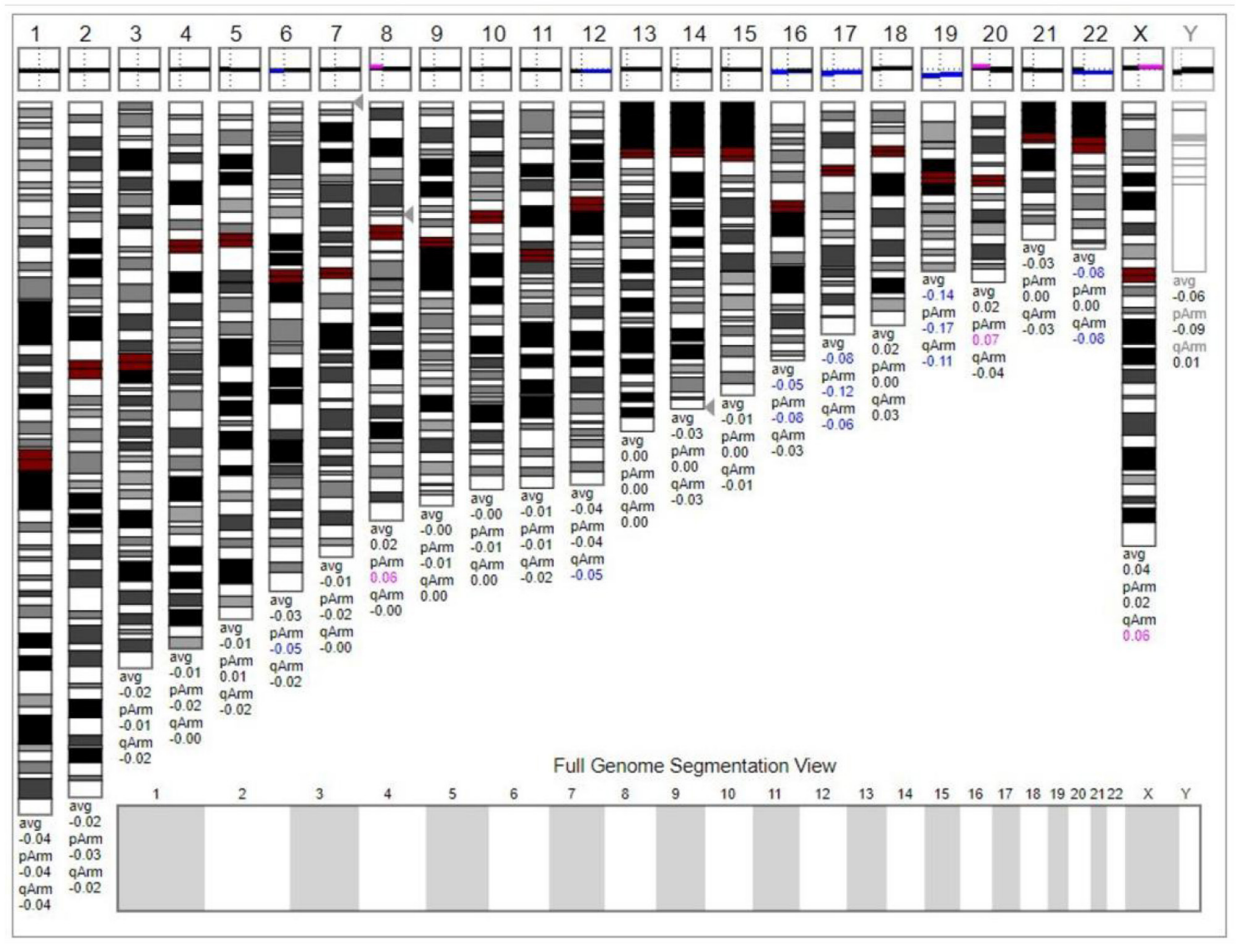
The chromosome microarray did not detect duplicates or microdeletions in the chromosome. This karyogram was generated by Genoglyphix v3.0 according to the microarray results. Triangle markers on the right side of the chromosomes indicate the alteration locus. Pink indicates duplication; blue indicates deletion, and gray indicates duplication or deletion excluded by Genoglyphix v3.0.

The chromosome karyotype analysis showed no obvious chromosome structure abnormality in the child. Further, we tried to detect duplicates or microdeletions in the chromosome through the chromosome microarray method, however, no positive findings were found, as shown in Figure 3.

### Whole-Exome Sequencing

**Figure 4:**
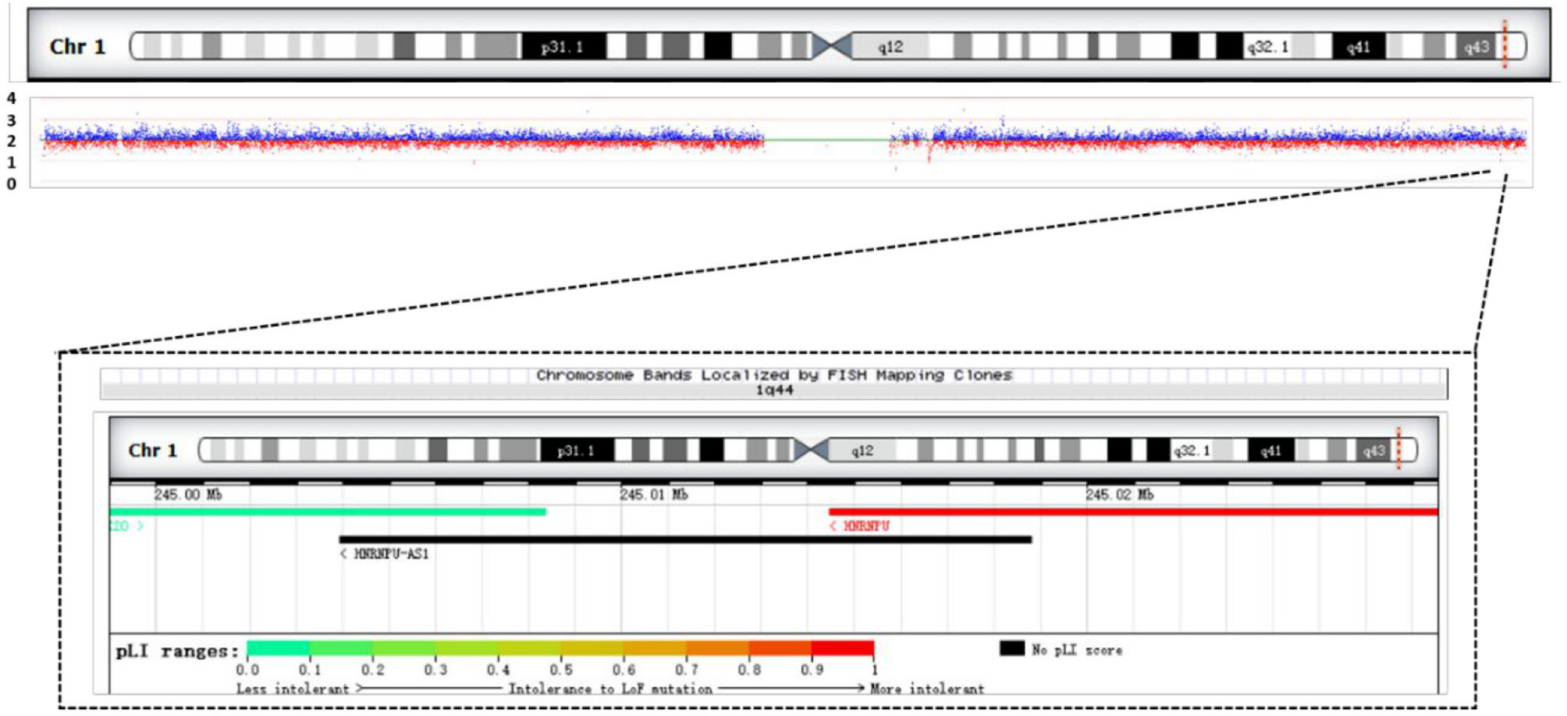
Results of CNV analysis based on exome trapping sequencing depth in the patient. It shows a 28.7 KB heterozygous deletion (chr1:244999016-245027609, hg19) in the 1q44 region. Overview of patient's chromosome 1 deletion and schematic diagram of the gene contained in the deletion region.

We performed SNP analysis of the total exome detection in our patient. The results showed no suspected pathogenic mutation sites. Included regions related to tics, mental and language delay, corpus callosum dysplasia, and polydactyly were detected. We performed CNV analysis based on capture copy number variation (CapCNV) on the patient's whole-exome detection results. The results showed that a 28.7 kb deletion was detected in the 1q44 region (chr1:244999016-245027609 ^*^ 1) containing two genes, as shown in Figure 4 and Table 1.

**Table 1:** The deleted protein coding genes in the 1q44 region.

**Gene**	**Phenotype**	**Inheritance**	**Gene function**
*COX20*	Mitochondrial complex IV deficiency	AR	The COX20 gene encodes a protein involved in the assembly of mitochondrial complex IV; it interacts with MTCO2
*HNRNPU*	Epileptic encephalopathy, early infantile, 54	AD	The HNRNPU gene encodes a highly conserved protein that binds RNAs and mediates different aspects of their metabolism and transport

### Fluorescent Quantitative PCR

**Figure 5:**
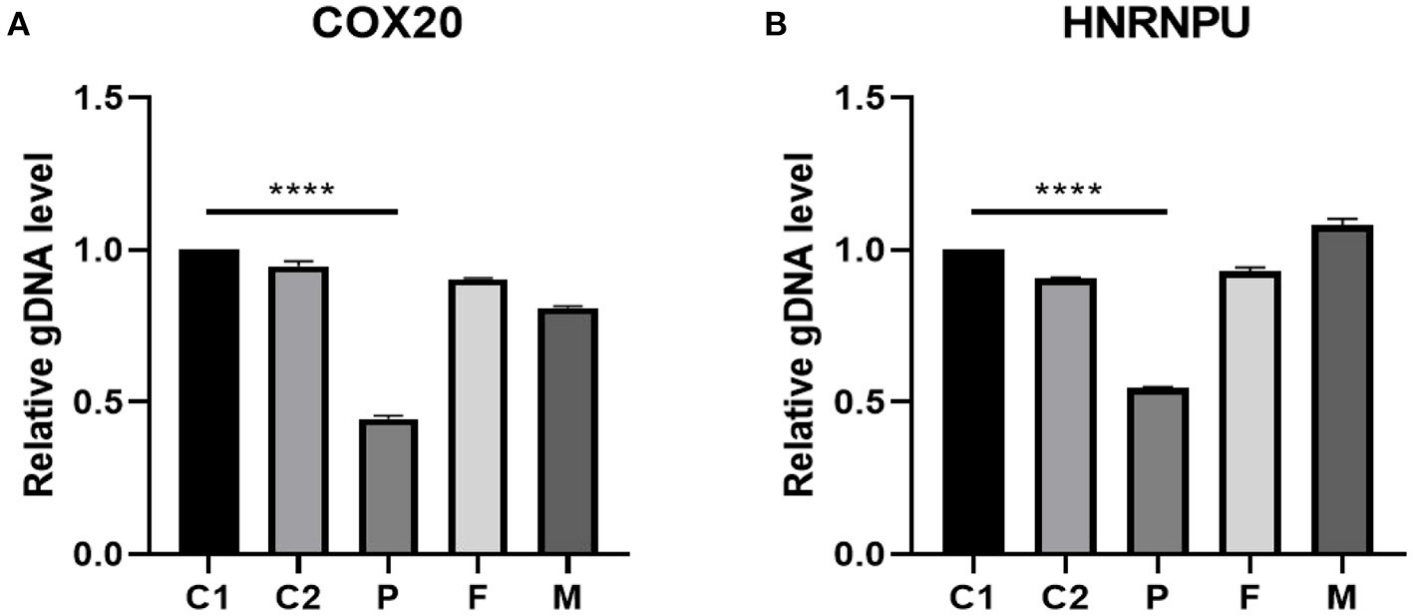
gDNA level validated by real-time fluorescent quantitative PCR in the 1q44 region. The results suggested that the patient had a heterozygous deletion in the COX20 **(A)** and HNRNPU **(B)** genes (relative quantification ratio close to 0.5). Additionally, the parents of the patient had no deletion in COX20 and HNRNPU, suggesting that this variant was a new variant. C1 and C2 stand for controls, P stands for patient, F stands for father, and M stands for mother. Differences were considered significant at *p* < 0.05. ****p < 0.0001.

We used the exome of the COX20 gene (ZBTB18; OMIM# 608433) and HNRNPU gene (OMIM# 602869) deletion region to design fluorescent quantitative PCR primers. The results indicated that haploinsufficiency was apparent in COX20 and HNRNPU (the relative quantification was close to 0.5), which was consistent with the whole exome's results. Additionally, the patient's parents had no deletion found in the COX20 and HNRNPU genes (relative quantification ratio close to 1), suggesting that this was a *de novo* variant as shown in Figure 5.

## Discussion

Deletion in chr1qter was widely considered to have strong relevance to neurodevelopmental diseases such as epilepsy and intellectual disability, which can be divided into proximal deletion (1q21-22q25), intermediate deletion (1q24-25q32), and terminal deletion (1q42-44). The first report was published in 1976 by Mankinen, which described a 3-year-old child who presented with microcephaly and epilepsy (Mankinen et al., 1976). Mankinen et al. used C-banding to detect chr1 in this girl. In 1981, Juberg suggested that the terminal deletion of the long arm of chr1 should be clinically diagnosed as a type of syndrome (Juberg et al., 1981). Roberts et al. then described the syndrome phenotype accurately, including psychomotor delay, growth delay, and facial dysmorphism (Smith et al., 1980). More and more cases of 1q deletion have been reported to improve CNV detection accuracy based on the development of genome microarray and other methods for detecting CNV.

Some studies have reported that AKT3, HNRNPU, and ZBTB18 are associated with the neurodevelopmental phenotype of the syndrome (Easton et al., 2005; Ballif et al., 2012; Thierry et al., 2012; Speevak et al., 2013). Easton et al. described a study of AKT3 knockout mice (KO) for AKT3 which resulted in a reduction of brain volume and the size of the corpus callosum, which suggested that AKT3 may be a candidate gene for ACC/MIC (Easton et al., 2005; Raun et al., 2017). Hill et al. and Boland et al. reported that the deletion of the 1q43q44 fragment was associated with MIC (microcephaly), ACC (agenesis of the corpus callosum), and SZR (seizures) (Doco-fenzy et al., 2006; Boland et al., 2007). They precisely located the key region for MIC and found out that if the AKT3 quantity were reduced to less than half normally located in this region, this would likely lead to MIC/ACC. However, van Bon et al. located AKT3 outside the critical region of ACC afterward, which ruled out the relevance between AKT3 and ACC. Meanwhile, Nagamani et al. demonstrated that AKT3 at low haploinsufficiency is not able to induce ACC, but capable of leading to MIC, suggesting that AKT3 is a strong candidate gene for MIC. However, Gai et al. reported an AKT3-exclusive deletion in a father and son pair where the father had a normal head circumference and the son had microcephaly (−1.9 SD). Despite the same genetic pathology, it indicates that the role of AKT3 in microcephaly is more complicated and may have incomplete penetrance (Gai et al., 2015). Nagamani et al. also found two new candidate genes for ACC, which are known as CEP170 and ZBTB18. ZBTB18 has also been reported to be a key candidate gene for MIC and general developmental delay in 1q43-q44 deletion syndrome (Nagamani et al., 2012; Raun et al., 2017). Dominik et al. considered that ZBTB18 was the most probable candidate gene for HCC/ACC and also had the possibility of incomplete penetrance (Westphal et al., 2017).

The link between the genotype and specific phenotype of 1q43-q44 deletion syndrome is still unclear due to the phenotypic heterogeneity, the small sample size in most of the studies, the large amount of data, and their complicated information. The results of the genetic and phenotypic analysis of this patient presented a microdeletion of the 1q44 region, which was covered with HNRNPU and FAM36A. With the results of studies about the minimal overlapping region published before, we compared the deletion region of our patient, which highly overlapped with the SZR-related region, leading us to the explanation of her epileptic phenotype (Hemming et al., 2016; Depienne et al., 2017; Yates et al., 2017). AKT3 was not found in her deletion region, which was consistent with the study that linked AKT3 with MIC since she did not show signs of MIC. According to the study of ACC in the past, they found that ZBTB18 had high relevance with ACC (de Munnik et al., 2014; Cohen et al., 2017; van der Schoot et al., 2018). She did present with agenesis of the corpus callosum, however, without the particular genetic lesion. The phenotype with ACC may be related to HNRNPU (Ballif et al., 2012). Depienne et al. have also documented that the loss-of-function of HNRNPU can cause neurodevelopmental abnormalities and that HNRNPU inadequate haploinsufficiency is one of the mechanisms of 1q44 deletion syndrome (Depienne et al., 2017). Carvill et al. suggested that haploinsufficiency of HNRNPU is associated with epileptic encephalopathy and intellectual disability (Carvill et al., 2013). HNRNPU has been detected in patients with psychomotor delay, growth delay, and facial dysmorphism. Caliebe et al. and Thierry et al. suggested that HNRNPU deletion is a possible cause of epilepsy and ID in patients with 1q43q44 deletion. A study cohort of patients with HNRNPU mutations by Yates showed that all patients had developmental delay and intellectual disability (ID), ranging from moderate to severe. Seizures were common and occur in the context of febrile episodes initially. And these patients always had dysmorphic features, such as long palpebral fissures, thin upper lip, prominent eyebrows, and overhanging columella (Caliebe et al., 2010; Thierry et al., 2012). De Kovel et al. mentioned a patient with early developmental delay, febrile seizures, which later became afebrile seizures (de Kovel et al., 2016). Our patient, as well, presented with a period of febrile seizures proceeding into afebrile seizures, refractory epilepsy, global developmental delay, and multiple dysmorphisms. Her clinical presence was matched with HNRNPU-associated phenotypes.

At the same time, the girl also presented with symptoms such as hypoplasia of the right eyeball, postaxial polydactyly, and amelogenesis imperfecta, which are difficult to explain by her missing region. We suggested that there might not be a simple one-to-one relationship between the 1q terminal deletion region and the clinical phenotype. The possibility that a single inherited phenotypic trait is controlled by multiple different genes should be considered. The key pathogenic genes identified in most previously reported cases might be the major genes for some clinical phenotypes, while other genes also affected partial clinical phenotypic traits. This might be the reason why some cases do not develop into a particular phenotype while they do carry the key genetic lesion.

The patient's clinical phenotypes were highly suggestive of chromosomal diseases, yet no abnormalities were detected by convention karyotype analysis and chromosome microarray techniques. Eventually, whole-exome sequencing combined with the capCNV technique was used to detect the 28.7 KB deletion in the chr1qter region, which was the smallest reported deletion at the time. Conventional cytogenetics karyotype analysis has a resolution of about 5–10 MB, and the ends of chromosomes often appear as unstained areas under an optical microscope. This limitation can lead to ignorance of small deletions that cannot be detected, which results in the “normal” chromosome karyotype of our patient. The chromosome karyotype analysis of this patient showed no abnormalities, and although the chromosome microarray can further detect smaller genomic deletions, the microdeletions reported here may not be detectable. The whole-exome sequencing method has the advantages of high resolution, high throughput, and high sensitivity. It can detect single-base mutations. The CapCNV analysis based on whole-exome sequencing can further improve the resolution and accuracy of CNV detection. These techniques allow us to further study the relationship between CNV and the phenotype trait and even set up the foundation of the pathogenesis of complex diseases.

## Data Availability Statement

The datasets for this article are not publicly available due to concerns regarding participant/patient anonymity. Requests to access the datasets should be directed to the corresponding author.

## Ethics Statement

The studies involving human participants were reviewed and approved by Children's Hospital of Nanjing Medical University. Written informed consent to participate in this study was provided by the participants' legal guardian/next of kin. Written informed consent was obtained from the individual(s), and minor(s)' legal guardian/next of kin, for the publication of any potentially identifiable images or data included in this article.

## Author Contributions

GZha and GZhe designed and performed the study. YT, HL, and WL wrote the draft manuscript. TH and GH collected the data. SK carried out data analysis and language revising. All authors approved the final manuscript for submission.

## Conflict of Interest

The authors declare that the research was conducted in the absence of any commercial or financial relationships that could be construed as a potential conflict of interest.
